# NK cell depletion promotes liver metastasis of lung cancer cells

**DOI:** 10.3724/abbs.2023266

**Published:** 2024-01-22

**Authors:** Pan Yu, Long Zhang, Jianhui Tian, Jiajun Liu, Zujun Que, Ge Li, Yiyang Zhou

**Affiliations:** 1 Clinical Oncology Center Shanghai Municipal Hospital of Traditional Chinese Medicine Shanghai University of Traditional Chinese Medicine Shanghai 200071 China; 2 Institute of Oncology Shanghai Municipal Hospital of Traditional Chinese Medicine Shanghai University of Traditional Chinese Medicine Shanghai 200071 China; 3 Dalian Hospital of Traditional Chinese Medicine Dalian 116013 China

Lung cancer is the malignant tumor with the highest morbidity and mortality in the world [1]. Metastasis is the main cause of death in lung cancer patients [2]. Cancer cells that are shed from the primary tumor tissue and enter the peripheral circulation are called circulating tumor cells (CTCs) [3]. Among them, tumor cells that reach and colonize distant target organs are named disseminated tumor cells (DTCs) [4]. Influenced by the microenvironment, immune surveillance, angiogenesis and other factors, DTCs have a weak ability to proliferate or even enter a dormant state [5]. Therefore, reducing proliferative activity or maintaining the dormancy of DTCs has emerged as a novel strategy to prevent lung cancer metastasis.

It is widely known that immune cells, such as T cells and natural killer (NK) cells, play an important role in controlling the development and progression of cancer metastases [6]. T cells rely on antigen presentation by dendritic cells to exert a powerful tumor cell killing effect, and well-established clinical treatment protocols are now available [7]. NK cells are the arm of the innate immune system with the capability to kill tumor cells, including antibody-dependent cell-mediated cytotoxicity (ADCC), the FASL/TRAIL pathway, and TNF-α secretion [8]. NK cells are the first line of defense against tumors and are not limited by antigen delivery, thus having strong potential to combat lung cancer metastasis. Liver metastasis occurs in 20% of patients with advanced non-small cell lung cancer and has a median survival of 4 months, representing a truly poor prognosis [ 9, 10]. In this study, the role of NK cells in the liver metastasis of lung cancer was evaluated by constructing a preclinical lung cancer metastasis model and exploring the potential mechanism.

To investigate the role of NK cells in lung cancer metastasis, a mouse model of lung cancer metastasis was constructed. Six-week-old male C57BL/6J mice were injected with mouse lung cancer Lewis cells (5×10 ^5^ cells) through the tail vein. Anti-asialo GM1 (Wako, Tokyo, Japan; 20 μg/mouse) was administered intravenously 1 day before and 3/7/11 days after tumor cell injection to deplete NK cells in mice. Three weeks later, the body weight ( Supplementary Figure S1A) and the proportions of NK cells in the peripheral blood and spleen ( Supplementary Figure S1B) were significantly reduced. In addition, the weights of the lung and liver were significantly increased ( Supplementary Figure S1C,D), indicating that the metastases of the lung and liver were more severe.

As shown in Figure 1A, the number of metastatic nodules on the lung surface of mice with depleted NK cells was significantly increased. All NK cell-depleted mice developed visible lung metastases, while only half of the mice in the control group developed lung metastases ( Figure 1B). Meanwhile, only one metastatic nodule was visible on the surface of the liver in the control group, while there were dense metastatic nodules on the liver surface of each NK cell-depleted mouse ( Figure 1C,D). Metastatic nodules in the lung and liver were pathologically confirmed by HE staining ( Figure 1E,F). Based on HE staining, K-Viewer software was used to calculate the areas of metastatic foci and the entire organ section separately, and the metastasis index was calculated as follows: lung metastasis index=tumor area/lung area×100%; and liver metastasis index=tumor area/liver area×100%). We found no statistically significant difference in the lung metastasis index between the anti-NK group and the control group ( Figure 1G). Meanwhile the liver metastasis index increased significantly after NK cell depletion ( Figure 1H). These results indicated that, compared to lung metastasis, NK cell depletion substantially facilitated liver metastasis in lung cancer mice.

**Figure 1:**
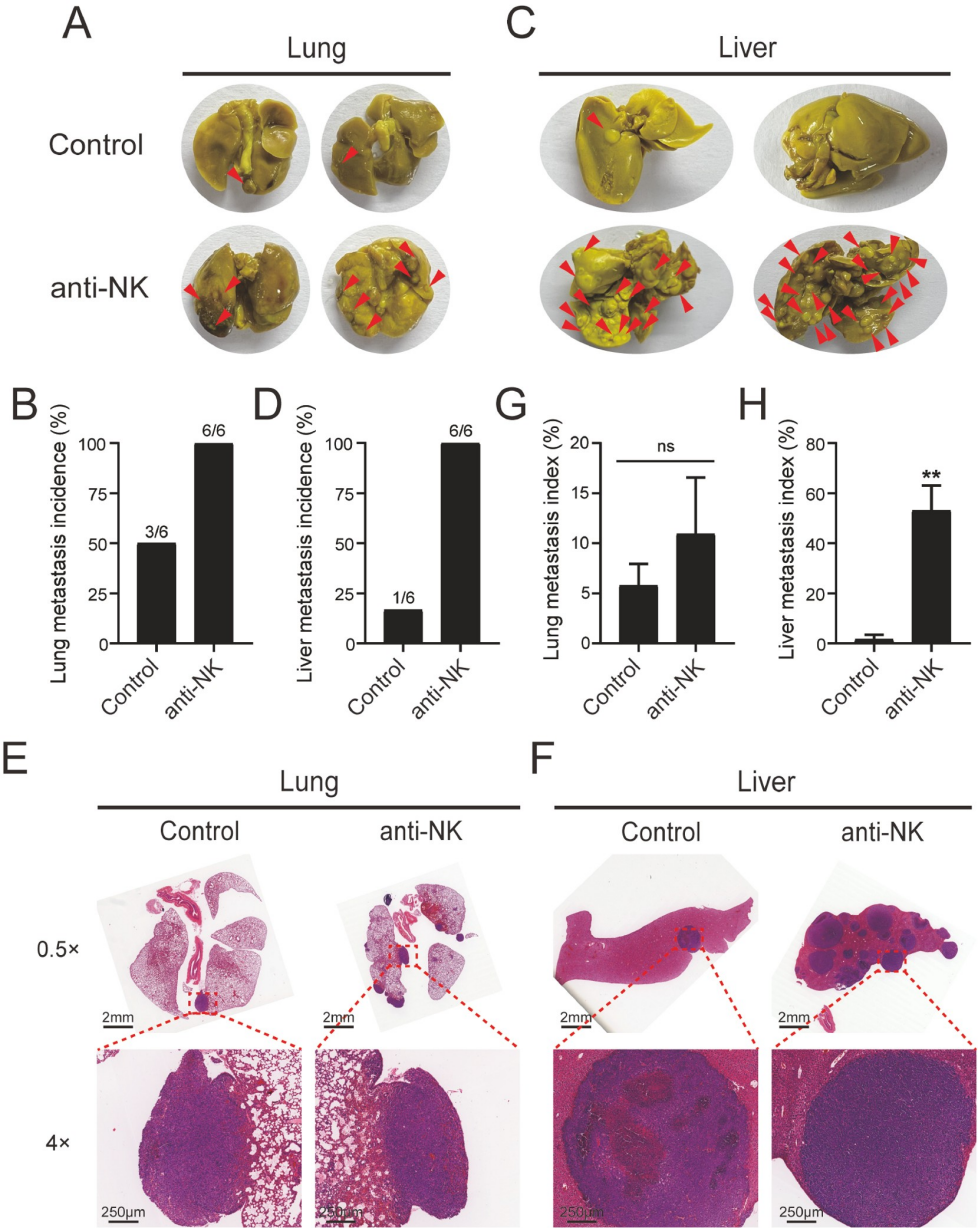
Figure 1 Depletion of NK cells promotes the liver metastasis of lung cancer in mice Six-week-old male C57BL/6J mice were injected through the teil vein with 5×105 Lewis cells and treated with anti-asialo GM1 (20 μg/mice in 100 μL PBS, i.v.) or the vehicle (100 μL PBS, i.v.) 1 day before and 3/7/11 days after tumor cell injection. Representative images of lung (A) and incidence of lung metastases (B) in mice. Representative images of liver (C) and incidence of liver metastases (D) in mice. Representative HE staining images of lung (E) and liver (F). Metastasis index of lung (G) and liver (H) in mice. Data are presented as the mean±SD (n=6 mice/group). **P<0.01 vs the control group. i.v., intravenously.

We found that significant liver metastasis occurred after a decrease in NK cell level. A previous study has revealed that the activation of hepatic stellate cells after liver injury leads to low proliferative activity and impaired function of NK cells, resulting in the development of liver metastases from breast cancer. In addition, NK cells sustain breast cancer cell dormancy in the liver through IFN-γ signaling, and the microenvironment in which dormant cells are located contains more IFN-γ ^+^ NK cells [11]. NK cells make up approximately 40% of the total lymphocytes in the human liver and 5%‒10% in mice. Obviously, patients with solid tumors susceptible to liver metastasis should have concomitant liver protection to prevent liver metastasis caused by proliferation and reactivation of dormant DTCs in the liver.

To further explore the function of NK cells in the lung cancer metastasis mouse model, the levels of TNF-α and IFN-γ in the serum were measured by ELISA. As shown in Figure 2A, there was no significant change in serum TNF-α between the two groups. Nevertheless, compared with the control group, IFN-γ in the serum of the anti-NK group was significantly reduced ( Figure 2B). Therefore, we speculated that NK cells might influence the proliferation of metastases by secreting IFN-γ. Then, NK cells and IFN-γ in liver metastases were stained by immunofluorescence. We found that large numbers of NK cells and IFN-γ gathered at the edge of metastases in the control group, while the anti-NK group did not ( Figure 2C). Next, the effect of NK cell depletion on the proliferation rates of lung and liver metastases was investigated by immunohistochemical staining. The percentage of proliferating cells expressing Ki-67 was significantly increased in the anti-NK group compared with the control group in the lung and liver ( Figure 2D–F). To further investigate the effects of IFN-γ on the proliferation of lung cancer cells, we inoculated a small number of Lewis cells on matrix gel to emulate the dormant state of DTCs. IFN-γ treatment started 1 h after cancer cell seeding and the medium was refreshed every day. The cells were imaged on day 6 after staining with an EdU-555 cell proliferation assay kit. As shown in Figure 2G, the proliferation of Lewis cells was significantly reduced after mouse IFN-γ treatment. Moreover, the proportion of EdU-positive cells, which represents the cell proliferative state, was significantly reduced. To examine whether human IFN-γ has the same effect, we used human lung adenocarcinoma circulating tumor cells (CTC-TJH-01). Similarly, human IFN-γ restrained the proliferation of CTC-TJH-01 and sustained its dormancy ( Figure 2H). These results demonstrated that NK cells play an important role in inhibiting proliferation and sustaining dormancy of DTCs by secreting IFN-γ, especially in the liver, and NK cell depletion promotes the proliferation of Lewis cells *in vivo*.

**Figure 2:**
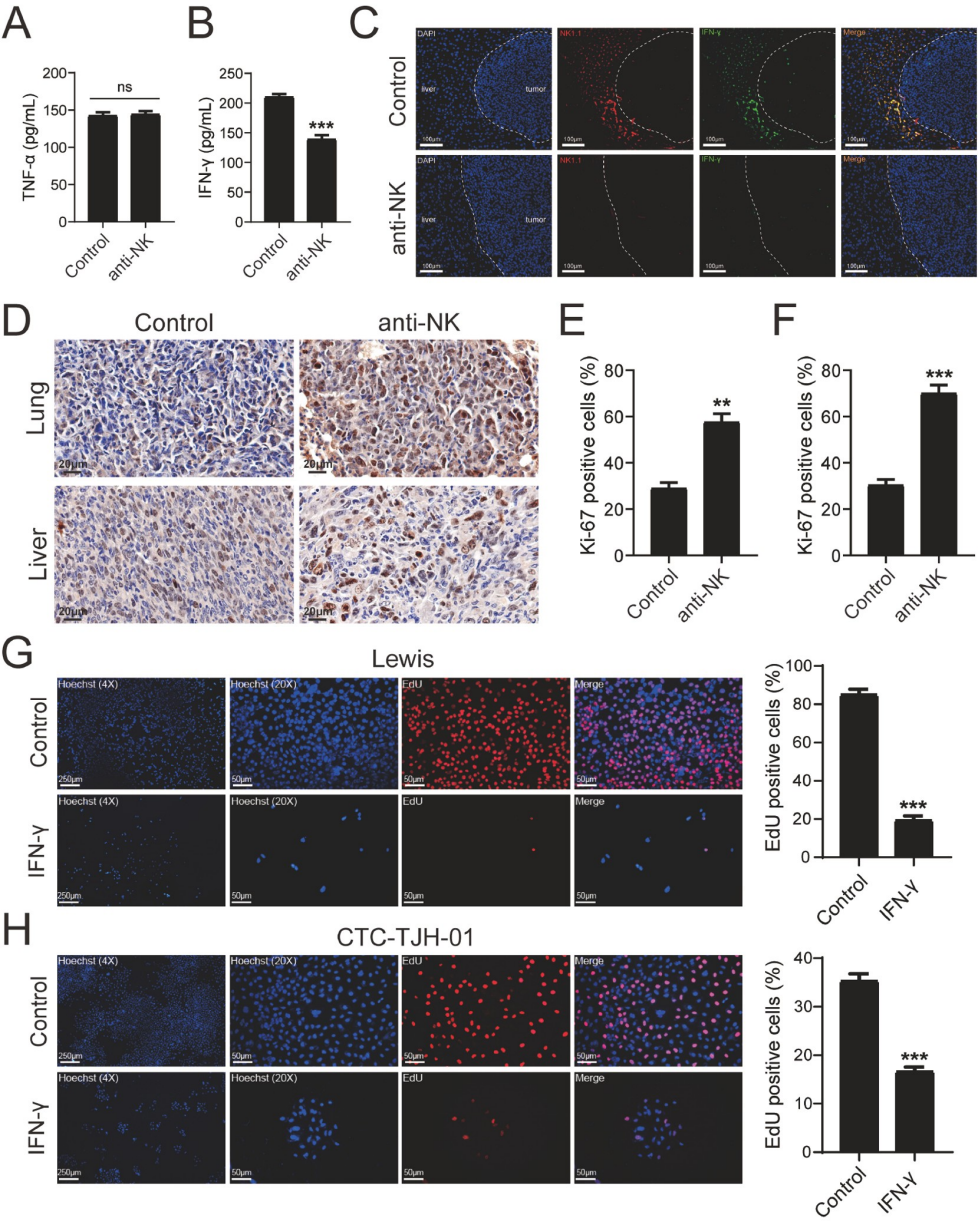
Figure 2 NK cells inhibit the proliferation of disseminated tumor cells by secreting IFN-γ The levels of serum TNF-α (A) and IFN-γ (B) in mice. (C) The expression of NK1.1 (red)- and IFN-γ (green)-positive cells in liver metastases stained by immunofluorescence. Dashed lines indicate the tumor margins. (D) Representative immunochemistry (IHC) images for Ki-67. Percentage of Ki-67-positive cells in lung (E) and liver (F) metastases. Data are presented as the mean±SD (n=6 mice/group). Representative images and quantification of EdU-positive Lewis cells (G) and CTC-TJH-01 cells (H) (red). The cells were seeded on Matrigel to emulate dormant DTCs and then incubated with fresh medium with or without IFN-γ (10 ng/mL) changed daily for 6 days. **P<0.01, ***P<0.001 vs the control group.

In summary, our study showed that liver metastasis is dramatically promoted by NK cell depletion in lung cancer mice. NK cells reduces the proliferation of Lewis cells in liver metastases and sustains DTC dormancy by secreting IFN-γ. In addition, we also established an animal model of liver metastasis from lung cancer that can be used for further mechanistic studies and new drug development.

## Supporting information

23321Supplementary_figure_S1

## Supplementary Data

Supplementary Data is available at *Acta Biochimica et Biophysica Sinica* online.
