# Supplementary material for: NK cell depletion promotes liver metastasis of lung cancer cells
Source: Acta Biochim Biophys Sin (Shanghai). 2024 Jan 22;56(2):323–6. doi: 10.3724/abbs.2023266 (PMC10984870; doi:10.3724/abbs.2023266)
Supplement: 23321Supplementary_figure_S1 [file 23321Supplementary_figure_S1.pdf]

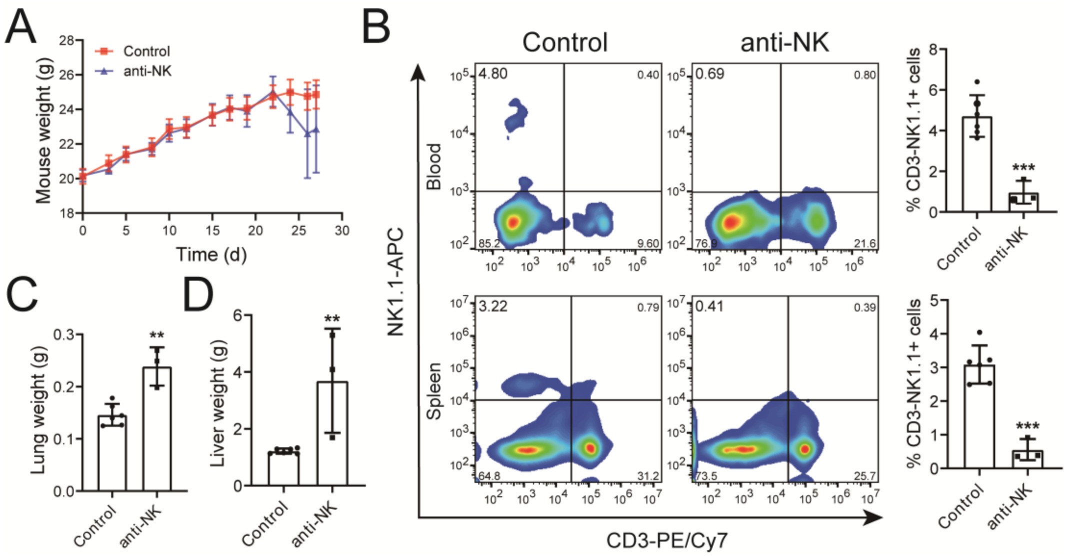

Supplementary Figure S1. Construction of NK cell depletion lung cancer metastasis mice model Six-week-old male C57BL/6J mice were injected intravenously with 5×10^5 Lewis cells and treated with anti-asialo GM1 (20 μg/mice in 100 μL PBS, i.v.) or the vehicle (100 μL PBS, i.v.) 1 day before and 3/7/11 days after tumor cell injection. (A) Change in mouse weight in the indicated groups. (B) Proportion of NK cells in peripheral blood and spleen of mice detected by flow cytometry. The lung (C) and liver (D) weight of mice in the indicated groups. Data are presented as the mean ± SD (*n*=6 mice/group). **P*<0.05, ***P*<0.01, ****P*<0.001 vs the control group. i.v., intravenously.
